# Could Western Attitudes towards Edible Insects Possibly be Influenced by Idioms Containing Unfavourable References to Insects, Spiders and other Invertebrates?

**DOI:** 10.3390/foods9020172

**Published:** 2020-02-11

**Authors:** Victor Benno Meyer-Rochow, Aimo Kejonen

**Affiliations:** 1 Department of Ecology and Genetics, Oulu University, SF-90140 Oulu, Finland; 2 Agricultural Science and Technology Research Institute, Andong National University, Andong 36729, Korea; 3 Geologian tutkimuskeskus, Itä-Suomen yksikkö, PL 1237, SF-70211 Kuopio, Finland; geologi.kejonen@gmail.com

**Keywords:** disgust, emotions, entomophagy, sociolinguistics, food choice, mirror neurons

## Abstract

It is known that idioms, proverbs, and slogans can become integrated into feelings like irritation, contemptuous attitudes, and even anger and disgust. Idioms making reference to insects, spiders, and other invertebrates occur in all languages, but they convey mostly negative content in people of Western cultural orientation. By analyzing a subgroup of insect and spider idioms related to food, eating, and digestion, the authors suggest that mirror neurons are activated in people that are exposed to the largely unfavorable content of such idioms. This could then lead the listener of such idioms to adopt the kind of negative attitude towards insects that is expressed in the idioms and to project it towards edible species.

## 1. Introduction

There is no single reason that explains why people would accept or reject insects as food. Vabø and Hansen [1] in connection with food preferences suggested that the latter strongly depended on the perceived healthiness of the food item, the price of the food item and convenience of obtaining it, its sensory appeal, the mood of the consumer as well as familiarity, and ethical concerns. Lensvelt and Steenbekkers [2] added to this list supply and demand, tradition, religious beliefs, etc., while Shouteten et al. [3] and Ghosh et al. [4] focused on sensorial, economic, cultural, and ethnic aspects. Peer pressure and socio-cultural perspectives were investigated by Menozzi et al. [5] and Tan and House [6], respectively, and disgust and neophobia in connection with Westerners’ disgust for eating insects formed topics of inquiries for La Barbera et al. [7] and Castro and Chambers [8]. Although researchers could show that the name, type, and role of an insect [9] can affect a species’ acceptance or rejection just like the image that an insect’s name creates in the consumer does [10], none of these as well as other investigators had previously considered that a rejection of insects as food could also have a linguistic background component. 

Idioms and proverbs, for example, occur in all languages and according to Casas et al. [11], constitute categories that “permeate languages at a much deeper level than what is usually taken for granted”. The same authors declare that idioms exert an “overriding influence” on society and according to [12] become integrated into popular consciousness to express feelings like irritation, contemptuous attitudes, and even anger and disgust [13]. Unsurprisingly, accepting the validity of this view, idioms and proverbs might then be interpreted as “natural decoders of customs, cultural beliefs, social conventions, and norms” [14] with a profound influence on experience and behaviour [15].

Turning now specifically to idioms that incorporate references to insects and other terrestrial arthropods like spiders, it has repeatedly been shown that amongst people with Western cultural backgrounds these idioms convey predominantly negative attitudes, but that in East Asia more positive attitudes prevail [16,17,18,19]. Thus, the question arises as to whether the negative insect idioms stemmed from a genuine, deep-rooted antipathy towards insects as well as terrestrial arthropods generally or whether the negative attitude has been nurtured and strengthened by references to such idioms that are explicitly negative with regard to these invertebrates.

What seemed pertinent in connection with this question is the recent realization of so-called mirror neurons in the human brain, discovered by Rizzolatti and Sinigaglia [20]. Such neurons are implicated in the ability of an individual to understand another individual’s emotion and to empathize by activating neuronal circuits, as when the receiving individual experienced the other person’s emotion herself or himself directly [21]. Originally seen in connection with visual or odoriferous stimulation, mirror neurons are now also thought to play a role in adopting emotions that are expressed figuratively, i.e., in idioms, as mirror neurons are located near the Broca area of speech development in the human brain and thereby can facilitate the learning process as well as verbal communication [21]. The emotional state of the ‘receiver individual’ according to Bastiaansen et al. [13] comes to resemble that of the ‘sending individual’, especially if a facial expression of disgust [22] accompanies the utterance of a particular idiom.

The aim of this paper has been to show that idioms containing negative references to insects and other invertebrates could possibly influence a person’s attitude, especially in connection with insects as a novel food item in Western countries. Therefore, this paper must not be seen as a comprehensive and detailed study on insect containing idioms and their effects on people, but is meant to serve as a ‘wake-up-call’ to conduct further studies into this question.

## 2. Materials and Methods

The target subgroup of insect, spiders, and other invertebrate-related idioms were those that show a relationship to food, eating, and digestion in the widest sense. Although a variety of such idioms from different languages were investigated by us, the idioms that we have most thoroughly examined belonged predominantly to the Finnish language for three reasons: firstly, Finns have largely been a rural people living in close contact with Nature and their language is therefore particularly rich in idioms that contain references to insects; secondly, the authors’ own familiarity with the language provided us with considerable insight into use and meaning of such idioms; thirdly, in agreement with other people of Western cultural backgrounds, Finns until very recently, did not consider insects and other terrestrial arthropods as edible, seeing in fact most of them as pesky and annoying creatures. 

Since Finnish idioms and proverbs have been the subject of several books, the ten most relevant books [23,24,25,26,27,28,29,30,31,32] were consulted by the authors in order to locate suitable idioms, i.e., those involving insects, spiders, and other invertebrates in connection with food and eating. The idioms were discussed with a small but unspecified number of colleagues, five students of approximately 20 years of age and four elderly relatives, who contributed four additional idioms (referred to as ‘other’). The research represents a qualitative study of the relevant literature but does not claim to be exhaustive. As with an earlier study on Korean insect idioms by Meyer-Rochow [19], we are presenting the idioms first in their original form, followed by their translation into English and, where deemed necessary, an explanation as to the idiom’s meaning and application in parentheses. Each Finnish idiom’s source, i.e., the book it was found in, is indicated at the end of each idiom. How the non-Finnish idioms were found is mentioned in connection with the language they were taken from. 

## 3. Results

Finnish language speakers may find some of the expressions and spellings unusual, because often idioms are connected with local dialects that somewhat differ from the standard Finnish. Moreover, urbanized Finns may not recognize some of the older idioms, especially those used by rural folk.

### 3.1. Finnish Idioms

1. Kyllä se kärsii, mitä tai järsii [23] = He is in pain, who is eaten by a louse (meaning either that a small thing can cause lasting pain, or that some pains need to be tolerated).
2. Täi lihalla elää, mies muulla ruuvalla [23] = A louse lives by eating flesh/meat, humans eats other foods (humans ought to be more selective in their food choices).
3. Täi aina lihhoo syö [23] = A louse is always eating flesh/meat (see above).
4. Ei sääsken saaresta paljon paisteta [23] = One foot of a mosquito isn’t much of a roast (lamenting the lack of food).
5. Kussa vähä kimalaisia, siinaä vähä hunajata [23] = Where there’s few bumblebees, there’s little honey (with few persons around to be involved in work, life cannot be sweet).
6. Jaakko paarmat kokoo ja keittää niistä hutun [24] = John collects the horseflies and cooks a horsefly-porridge (someone who hasn’t got anything better to eat than poor food).
7. Sirkan reisi ja kirpun hanta koyhan miehen rasjas on [24] = The thigh of a cricket and the tail of a flea are in the food box of a poor man (there is not much else available to eat; expressing a severe lack of good food).
8. Tyyristä kärpäsen mesi [25] (kärpänen = fly, but can also be a bee in western Finland like here) = The honey of a fly is expensive (an item, of food perhaps, that is very rare and not easy to obtain by an ordinary person).
9. Minä tome, toucan ruoka, matoni pala maanalainen [25] = I am dust, the food of worms (maggots), the worms’ underground bread (a very negative view of one’s existence).
10. Yksin tehty työ on kun tervaa, kaksin kun hunajaa [26] = Working alone is tar, working together is honey (cooperation makes things easier).
11. Läksin mina kesäyömä käymään, sano kiljupytty muurahaismaättäässä [26] = I begin to “käydä”, here = to ferment, in a summer night, the barrel full of strong home-made beer it says in the anthill (meaning is a little unclear: perhaps to feel relaxed).
12. Ei niin pientä matoo, ettei hiukka rasva [26] = There is no worm so little as not to have a little fat (sarcastic and to mean that even poor food can be acceptable).
13. Niin on koyhä jottei ole täitää [26] = So poor that she/he does not even have lice.
14. Kiitoksia, sano Laurikainen, kun otti madon maasta ja pani suuhunsa [26] = Thank you says Laurikainen as he takes a worm from the earth (grateful for even the poorest food).
15. Sillä välin lapsen nälkä tulee, kun kärpänen pirtin yli lentää [27] = A child is getting hungry as soon as a fly flies over your house (there is so little nourishment at home that even a fly is seen as a desirable food item).
16. Kaunis kakku päältä nähden, mutta jos lie sirkkoja sisällä [27] = The cake may look beautiful, but there could be crickets (grasshoppers) inside (you could be deceived to believe that something is nice by the way it looks).
17. Hyttynen on niin kesy, että heti syö kädestä [other] = The mosquito is so tame, that it eats from your hand at once (sarcastic, meaning that something seemingly harmless, turns out be villainous and exploiting).
18. Parempi mato omenassa kuin puolikas puremassa [other] = Better a whole worm in an apple than half of the worm in the bitten place (jokingly refers to that you would have consumed half a worm, if you see the other half still in the apple).
19. Ei madon syöma makee, eikä hiiren syömä hipee [other] = Worm eating is not sweet and mouse nibbling is not good (having little to eat is not funny).
20. Mehiläinen meidän lintu, herhiläinen hiiden lintu [other] = Bee is our bird, hornet is the Devil’s bird (expresses dislike of hornets as evil in comparison with something good like bees).
21. Lisänä rikka rokassa, hämähäkki taikinassa [28] = More means a rock in the soup and a spider in the dough (sarcastic and to mean one should not expect too much and that even something as small as a spider can be an addition).
22. Ole on omituinen kuin olisi perhosia vatsassa [28] = Living is strange like having butterflies in your stomach (an expression of worry, anxiety, or nervousness).
23. Sirkan reisi ja paarman jalka köyhän aitasta löytyy [28] = The thigh of a cricket and the foot of a horsefly are in a poor man’s storehouse (there is not much available to eat; no proper food).
24. Kirpum potku ei paljoo tunnu [29] = Cooking a flea does not feel much (a small amount of food does not fill your stomach).
25. Paskakakkokim makosta haukatas sontiaisen suulla [29] = Even shit is good to bite, if you have the mouth of a dung beetle (you are prepared to eat the worst food).
26. Se on niin nuuka et se kirputki halkasee ja pitää itte molemmat puolet [30] = She/he is so stingy that she/he cuts the flea into two pieces and keeps both to herself/himself (someone in such need of nourishment that she/he would not even be willing to share with someone half a flea as food).
27. On niin ahne ett otais kirpultaki kolmppari koippei [30] = So greedy that she/he takes all three pairs of legs from a flea (someone in such need of nourishment that she/he would not even be willing to share one leg of a flea as food with somebody else).
28. Ain se avuks on ikä kärväne kaalfaris [30] = It is always as much help as a fly is meat in the cabbage casserole (the help rendered by someone is worth almost nothing).
29. On niinku kärväne siirapis [30] = To be like a fly in syrup (to be in some sort of serious trouble).
30. Käy niinko mato raatoo [30] = To eat like worms eat the carcass (here: mato = fly larva) (to be non-selective when it comes to food and even accept poor/disgusting food).
31. Meni ku mato rasvonee [30] = She/he went like a worm with its fat (someone did not share or leave anything useful).
32. Syä kun hevonen ja paskantaa kun peronen [30] = Eat like a horse and shit like a butterfly (someone who received a lot of support, but produced a meagre result).
33. Nielöö kun variksenpoeka sittapörrijä [30] = To swallow like a young crow swallows door beetles (when someone eats too much and too hurriedly not caring what she/he’s eating).
34. On vatta nin täysi että täin päälla tappas [30] = The stomach is so full, that it could easily kill a louse in it (someone has eaten very well and is satisfied).
35. Syöö kun hevone ja tekköö kun täe [30] = Eat like a horse and work like a louse (a lazy person that works little, but consumes much food).
36. Kun sirkka pääsee suurukselle, niinse laulaa [30] = When a cricket sits at the dinner table it is singing (if there is food around, people are happy).
37. Vennyy kun vesimato piimäsä [30] = To lie or stretch out like water worm in sour milk (a lazy and relaxed person).
38. Niin nuuk, et pannee kirpun poikki ja pittää molemap päätitte [31] = So greedy that she/he cut the flea into two pieces and kept both pieces to herself/himself personally (someone in such need of nourishment that she/he would not be willing to share with someone even half a flea as food).
39. Vattuvaha se on vatun matokii [32] = A raspberry worm is only a part of the raspberry (if one likes something, one needs to accept the problems that come with it).
40. Näläkästä se torakka sillon siälii kun mökinseinästä vellipataan tipahtaa [32] = The cockroach is feeling pity for poor people, when it drops from the wall into the gruel pot (even an insect like the cockroach can be a welcome addition to the food of poor people, because they have nothing much else).

### 3.2. Non-Finnish Idioms

To show that Finnish is not the only language that possesses idioms in the category of insects and spiders related to food, eating, and digestion, a few examples shall be given of such idioms of other languages.

Lithuanian (L. Panavaite, pers. comm.):
Alkanam šuniui ir grambuolys mėsa = a hungry dog sees a cockchafer as meat.

English [33]:

1. Flies come to feasts unasked.
2. A louse in the cabbage is better than no meat at all.
3. When the bee sucks, it makes honey; when the spider, it’s poison.

German [34]:

1. In der Not frisst der Deubel (= Teufel) Fliegen = in times of need the Devil consumes flies (if there is nothing else to eat, then like the Devil one would even accept flies as food).
2. Besser eine Laus im Topf als gar kein Fleisch. = Better a louse in the pot than no meat at all.
3. Da ist der Wurm drin= There’s a worm in it (it can be maggot or caterpillar) (something is not right; something is not ’kosher’).
4. Der/sie macht‘n Gesicht als haett er‘ne Spinne gefressen. = He makes a face as if he’d swallowed a spider (a person’s expression when something unpleasant has occurred to him/her).

Korean [19]:

1. 구데기 무서워 장 못담굴가 = Goodeogi mooseoweo jang motdamgeunda (one can make soybean paste sauce even in the presence of maggots).

Japanese [18]:

1. 蓼食う虫も好き好き Tade kuu mushi mo sukizuki. Literally: There are even bugs that eat knotweed (tastes differ and that there’s no accounting for taste).
2. 蟻 の 甘き に 就く が ごとし Ari no amakini tsukuga gotoshi. Literally: ants stick to sweet things (people gather together where there is something to be got).

Although assigning idioms to categories designated as ‘negative’, ‘neutral’, and ‘positive’ is a subjective exercise that is likely to show some variation depending on who makes the distinction, our view is that of the 40 Finnish idioms related to insects and eating, feeding and food, 20 contained predominantly negative, 9 neutral, and 7 positive information (idioms with seemingly identical meaning, but slightly different wording were not counted separately). Clearly negative are idioms like “Kirpum potku ei paljoo tunnu” and “On niinku kärväne siirapi” while “Kussa vähä kimalaisia, siinaä vähä hunajata” and “Yksin tehty työ on kun tervaa, kaksin kun hunaja” are seen as neutral and positive, respectively. Although we did not have complete comparative lists of insect idioms in connection with food and eating from other languages, what we can nevertheless state with confidence is, that it is the negative content which is always more dominant in Western than Eastern cultures. One idiom each from Korea and Japan has served as an example of the more positive attitudes towards insects in these countries.

## 4. Discussion

We need to stress that in Finnish and other languages there are many more idioms referring to insects, spiders, and worms than those we mention in this article. We deliberately selected only idioms that had some connection with food, eating, and digestion. It was, after all, our intention to demonstrate that the use of these figuratively explicit idioms could affect the attitude of people towards accepting insects as food. Therefore: did our study provide evidence for a role of insect idioms in the mindset of people with Western cultural backgrounds?

Most people would agree that if a happy and smiling one-year-old infant sees and hears another, unrelated baby cry, the mood of the former, happy infant will change and it may also start to cry for no apparent reason other than seeing and hearing another baby cry. Likewise, as Stadler [35] explains, when a customer happens to phone a shop owner and talks with an angry voice (irrespective of the call’s content (our addition)), it will affect and change the mood of the recipient. Emotions are copied by mirror neurons and learning is not involved. That repeated emotional stimulation can ultimately alter the emotional right hemisphere of the brain and lead to a deeper unconscious reaction in connection with the appropriate stimulation has originally been postulated by Tsunoda [36], who went as far as claiming that the brains of Japanese people differed from those of Westerners in regard to linguistic, musical, and aesthetic perception because of the greater appreciation of insects by the Japanese. 

Duda and Brown [37] thoroughly investigated lateral asymmetry of positive and negative emotions and Schapkin [38] confirmed hemispheric asymmetries in connection with emotional words. Holtgraves and Felton [39] studied hemispheric asymmetries in relation to the processing of negative and positive words and Beraha et al. [40], a year later, examined the hemispheric asymmetry for affective stimulus processing. Finally, Gainotti [41] very recently reviewed the evidence for the role of the right hemisphere in emotion processing. It therefore appears highly likely that frequent exposure to idioms expressing a negative attitude towards consuming insects as food can indeed lead to long-lasting effects and a habitual rejection of insects as a food item. Idioms and proverbs, after all, have been credited with an ability to affect the experience and the behaviour of humans [15].

Given that idioms with references to insects and spiders in Western cultures are predominantly negative, but less so in Eastern cultures [16,17,18,19,42], there could therefore be one until now overlooked component of the reasons why people with Western cultural orientation hesitate to accept edible insects. Obviously, semantic and scientific knowledge (of the insect mentioned in an idiom) can “affect the outcome of the degree of mirror” [13], irrespective of a person’s dislike or even disgust triggered by the person’s exposure to the insect idiom. Rational thoughts and learning can override the inherent and unconscious reaction mediated by mirror neurons. A person familiar with cockroaches, knowing the latter do not transmit or carry diseases will in all likelihood be displaying less disgust than someone who knows little more than that the insect in question is a pest, perhaps clumping it together with lice, fleas, and irritating bugs. Thus, educating the public that the vast majority of all insects represents beneficial species would be a step in the right direction. Phasing out negative insect idioms could also be helpful since it has been shown after Citron et al. [43] that negative idioms are rated as more arousing than positive ones, in line with results from single words. 

An aspect not to be ignored is ‘familiarity’ with insects and other arthropods. Urbanization certainly leads to an increasing alienization regarding insects and one consequence of this is that nowadays fewer people know and use idioms that make reference to insects and other arthropods as seen especially in the cases with English and German speakers when compared, for example, with the Japanese [18]. This, however, does not repudiate our suggestion that insect idioms did and still do affect the attitude with which people contemplate insects and spiders, for firstly, historically many more insect and spider idioms were in use than is now the case [44] and secondly, as with taboos [45], once prejudices and antipathies become established, it takes a very long time before they are abandoned. And in the same vein, once a food habit has taken root, it is difficult to eradicate [46]. 

## 5. Conclusions

In conclusion, to establish insects as a regular food item (even if it is simply as a component of some other food) in countries that in the distant past had given up the consumption of insects and other arthropods and now enjoy no shortage of other food items, is likely to be an uphill struggle. A number of reasons have been documented and predictions on the intentions of eating insect-based products have recently been formulated [47], but a possible effect of idioms and proverbs in shaping consumers’ attitudes towards edible insects and other arthropods has never before been considered as a possible factor—even though the power of words and the role of mirror neurons in connection with emotions has received considerable attention. New idioms appear all the time [48] and perhaps coining some like “forget about the pork when there’s a cricket on your fork” or “mealworms and spaghetti is food that makes you happy” can help change attitudes!
